# Cerebrospinal Fluid Biomarkers Profiling in Older Brazilians reveals that CSF leptin is associated with obesity but not with cognitive decline

**DOI:** 10.1002/alz70856_101317

**Published:** 2025-12-25

**Authors:** Guilherme B. de Freitas, Felipe K. Sudo, Bart Vanderborght, Felipe C. Ribeiro, Luis E. Santos, Fernanda G. Q. Barros‐Aragão, Fabrício A. Pamplona, Mychael V. Lourenco, Douglas P. Munoz, Paulo E. Mattos, Fernanda Tovar‐Moll, Sergio T. Ferreira, Fernanda Guarino De Felice, IDOR Memory Clinic Initiative

**Affiliations:** ^1^ Queen's University, Kingston, ON, Canada; ^2^ D'Or Institute for Research and Education, Rio de Janeiro, RJ, Brazil; ^3^ Federal University of Rio de Janeiro, Rio de Janeiro, Rio de Janeiro, Brazil; ^4^ Federal University of Rio de Janeiro, Rio de Janeiro, RJ, Brazil; ^5^ Oswald Cruz Foundation (FIOCRUZ), Rio de Janeiro, RJ, Brazil; ^6^ Universidade Federal do Rio de Janeiro, Rio de Janeiro, RJ, Brazil

## Abstract

**Background:**

Forty‐five percent of dementia cases are potentially preventable through changes in individuals’ lifestyles (for example, reducing sedentarism) or by treating associated disorders (such as hypertension and obesity). However, there is no consensus on the global importance of CSF analytes and these potential lifestyle risk modifiers (LRM) for dementia onset, ultimately impacting diagnosis, prognosis, and efficient treatments. We aim to identify potential CSF therapeutical targets associated with dementia and its LRMs.

**Method:**

Our cross‐sectional study included individuals cognitively unimpaired ([CU] N = 25, 67.8 ± 4.8 y/o), and with cognitive decline ([CD] *N* =  37, 73 ± 6.5 y/o). 28 CSF analytes candidates were profiled and included in our dataset, along with LRM (hypertension, obesity, physical exercise, insulin resistance, dyslipidemia). Feature selection through nested logistic regression (LR) models regularized by elastic net was employed to select optimal predictors for CD and associated LRM. Linear regression models were built with selected CSF analytes to evaluate their direct impact on LRM and CD.

**Result:**

CSF Aβ42/Aβ40, LXA4/cysLT (Lipoxin A4/cysteinyl leukotriene, oxytocin, age, and obesity were identified as predictors for CD, and subsequently only CSF leptin for obesity. The direct impact of obesity, age, and CD in the selected CSF analytes was estimated by regression models. Lower ratios of CSF Aβ42/Aβ40 and LXA4/cys‐LT, along with higher CSF oxytocin levels, are associated with CD, and obesity was inversely correlated with cognitive decline. However, there is no association between CSF leptin levels and CD, as leptin is only associated with obesity and age.

**Conclusion:**

Obesity was primarily related to changes in CSF leptin and negatively associated with CD. However, the effects of obesity in CD decline are not associated with CSF analytes evaluated.

